# The Time Course of Markers of Neutrophil Extracellular Traps in Patients Undergoing Revascularisation for Acute Myocardial Infarction or Stable Angina Pectoris

**DOI:** 10.1155/2016/2182358

**Published:** 2016-12-15

**Authors:** Ragnhild Helseth, Svein Solheim, Harald Arnesen, Ingebjørg Seljeflot, Trine Baur Opstad

**Affiliations:** ^1^Center for Clinical Heart Research, Department of Cardiology, Oslo University Hospital Ullevål, Oslo, Norway; ^2^Faculty of Medicine, University of Oslo, Oslo, Norway

## Abstract

*Background and Aims*. Neutrophil extracellular traps (NETs) have been identified in acute myocardial infarction. We assessed the time profile and association with infarct size for NETs markers in ST-elevation myocardial infarction (STEMI) and stable angina pectoris (AP).* Methods.* In 20 patients with STEMI and 10 with AP undergoing percutaneous coronary intervention (PCI), blood samples were collected before PCI (only AP group) and after 3 and 12 hours, days 1, 3, 5, 7, and 14 for measurement of NETs markers.* Results*. Double-stranded deoxyribonucleic acid (dsDNA) and nucleosome levels were higher in STEMI than AP until day 3 and 12 hours (*p* < 0.03, all). DsDNA declined after day 5 in both groups (*p* < 0.04, all), while nucleosomes declined until day 3 only in the AP group (*p* < 0.05, all). DsDNA correlated with peak troponin T and creatine kinase MB (CKMB) at day 5 (*r* = 0.48, *p* = 0.03, both) and with MRI-measured infarct size at days 5 and 7 (*r* = 0.61, *p* = 0.01 and *r* = 0.52, *p* = 0.04, resp.), while nucleosomes correlated with infarct size at day 5 (*r* = 0.58, *p* = 0.02).* Conclusions*. High levels of NETs markers were observed in STEMI shortly after revascularisation and were partly associated with infarct size. The decline thereafter in both groups indicates a role for NETs in both acute and chronic atherothrombosis.

## 1. Introduction

Neutrophil cell activation in acute myocardial infarction (MI) has lately gained attention. Quantitative amounts of neutrophils and several neutrophil granule proteins are suggested to be predictive of infarct size, left ventricular ejection fraction (LVEF), new cardiovascular events, and death after acute MI [1–6]. A decade ago, it became evident that neutrophils upon activation are able to release parts of their nuclear content with residing neutrophil granule proteins into the extracellular space to form spindle-like networks called neutrophil extracellular traps (NETs) [7]. Although NETs initially were thought to have their main role in infectious diseases, ensuring entrapment of microorganisms in areas with high concentrations of antimicrobial proteins [7], NETs have lately been identified in coronary artery disease (CAD) [8–10].

In acute MI, high levels of circulating cell-free deoxyribonucleic acid (DNA), a surrogate marker of NETs, have been reported [11–15] and also linked to infarct size [8, 13, 15]. NETs are also suggested to be present in coronary thrombi [8, 9], in mural atherosclerotic plaques [16, 17], and in stable angina pectoris (AP) where they further seem to serve as a predictor of coronary artery severity and risk of new coronary events [10]. By the seemingly wide-ranging prothrombotic properties like platelet entrapment and activation [18], activation of the coagulation system [19, 20], and inhibition of fibrinolysis [21], NETs are potentially important players in the pathogenesis of atherothrombosis.

The dynamic profile of circulating NETs in the acute and subacute phase of ST-elevation MI (STEMI) has not been reported in detail previously. In this study, we aimed to explore the time profile of the circulating surrogate markers of NETs double-stranded deoxyribonucleic acid (dsDNA) and nucleosomes (DNA-histone complexes) in patients with STEMI or stable AP undergoing coronary angiography with percutaneous coronary intervention (PCI). The time profiles of myeloperoxidase (MPO) and pentraxin 3 (PTX3), well-known neutrophil cell granule proteins, were also investigated in order to explore whether these proteins could be reflected in the measured markers of NETs. We further assessed whether levels of dsDNA, nucleosomes, and MPO were related to indices of myocardial injury and left ventricular function.

## 2. Material and Methods

### 2.1. Study Design

Thirty patients with CAD, 20 with STEMI, and 10 with stable AP, admitted to Oslo University Hospital (OUS) Ullevål, Norway, undergoing successful revascularisation with PCI, were included. Details of the study design have been reported previously [22]. In brief, inclusion criteria in the STEMI group were characteristic clinical symptoms, electrocardiographic ST-elevations, and angiographic verification of coronary artery occlusion, while characteristic clinical symptoms and CAD angiographically suitable for PCI were inclusion criteria in the AP group. Exclusion criteria in both groups were previous transmural infarction, cardiogenic shock, and serious comorbidity. All patients were medically treated according to current guidelines and gave written informed consent for study participation. The study protocol was approved by the Regional Committee for Medical Research Ethics and conforms to the ethical guidelines of 1975 Declaration of Helsinki.

### 2.2. Blood Sampling

Blood samples were collected by standard venipuncture immediately before PCI in the AP group and after 3 and 12 hours, days 1, 3, 5, 7, and 14 in both groups. All blood samples from day 1 and further were obtained in fasting state before intake of morning medication. Routine analyses were obtained by conventional methods. Serum was prepared by centrifugation within 1 hour at 2500 ×g for 10 min and EDTA plasma was prepared by centrifugation within 1 hour at 2500 ×g for 20 minutes at 4°C, both stored at −80°C until analysed.

### 2.3. Laboratory Analyses

Levels of dsDNA and nucleosomes were quantified in serum by use of Quant-iT Picogreen dsDNA Assay # P11496 (Invitrogen, Carlsbad, CA, USA) and ELISA Cell Death Detection kit # 11774425001 (Roche Diagnostics, Indianapolis, USA), respectively. Optical density values in the nucleosome assay were normalized to an internal positive control and expressed as arbitrary units of nucleosomes per milliliter (NU/mL). All related samples from one patient and samples from both groups were analysed on the same plate in order to reduce the significance of interassay variability. Levels of MPO were measured in EDTA plasma by ELISA (Mercodia AB, Uppsala, Sweden). Interassay coefficient of variation (CV) for the measurements of dsDNA, nucleosomes, and MPO were 2.5%, 28.7%, and 6.3%, respectively.

Myocardial injury was assessed by peak serum levels of troponin T (reference value < 0.03 *μ*g/L) and creatine kinase MB (CKMB) (reference value < 5 *μ*g/L) and by gadolinium late contrast enhancement technique based infarct size (%) measured by magnetic resonance imaging (MRI) after 6 weeks. Left ventricular function was assessed by left ventricular ejection fraction (LVEF) by MRI after 6 weeks. MRI measures were available in 16 patients.

### 2.4. Statistical Analyses

As the majority of variables were skewly distributed, nonparametric statistics were used throughout. Group differences were assessed by Mann-Whitney *U* Test or Fisher's Exact Test as appropriate, while overall change within a group and change between two time points within a group were assessed by Friedman Test and Wilcoxon Signed Rank Test, respectively. Correlation analyses were performed by Spearman rho. Due to the hypothesis generating nature of the study, Bonferroni corrections for multiple comparisons were not performed. The level of significance was set to *p* < 0.05. All statistical analyses were performed by SPSS software package, version 23.0.

## 3. Results

Patient characteristics at baseline are shown in Table 1. Beyond more established CAD in the stable AP group and thus more frequent use of secondary prophylactic cardiovascular drugs, the groups were comparable.

### 3.1. The Time Profile of Circulating Markers of NETs

#### 3.1.1. Double-Stranded Deoxyribonucleic Acid (dsDNA)

**Figure 1:**
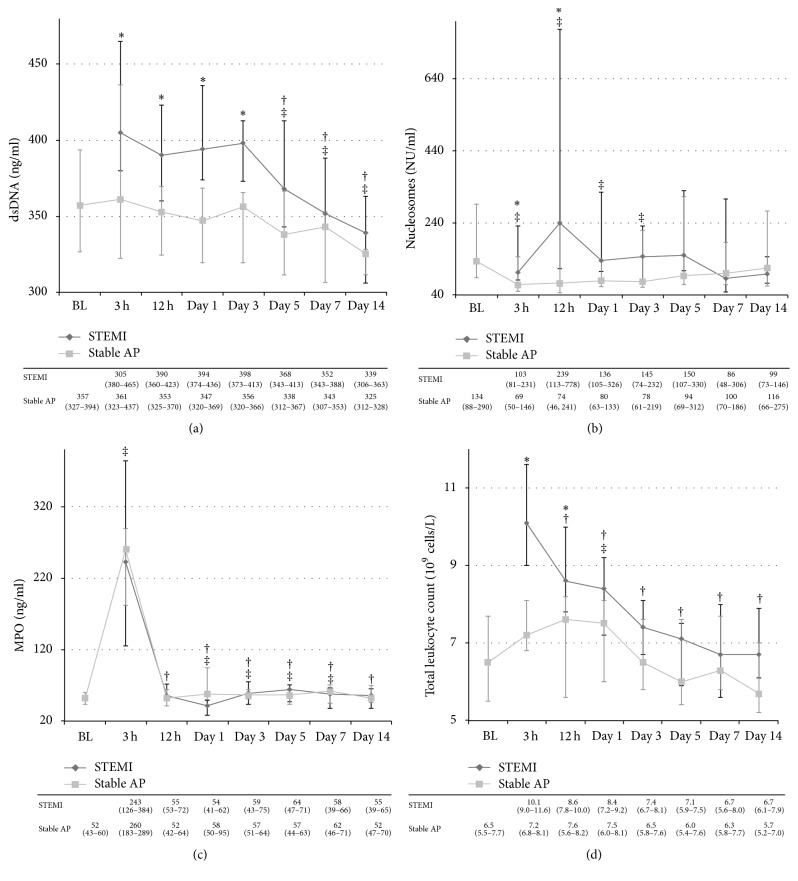
Time profiles of the NETs markers, myeloperoxidase, and leukocyte count. (a) Double-stranded deoxyribonucleic acid (dsDNA), (b) nucleosomes (DNA-histone complexes), (c) myeloperoxidase (MPO), and (d) total leukocyte count. The various time points (*x*-axis) and levels (*y*-axis) in the ST-elevation myocardial infarction (STEMI) and stable angina pectoris (AP) groups, as well as between- and within-group comparisons. Values are given as median (25–75 percentiles). BL: baseline. ^*∗*^ *p* < 0.05 for between-group difference at the various time points. ^†^ *p* < 0.05 for within-group difference from 3 hours in the STEMI group. ^‡^ *p* < 0.05 for within-group difference from baseline in the stable AP group.

Levels of dsDNA were significantly higher in the STEMI group than in the AP group at all time points until day 3 (*p* < 0.03 for all) (Figure 1(a)).

Within both groups, a significant overall change in dsDNA levels was observed (*p* ≤ 0.02), with values declining from 3 hours in the STEMI group and from baseline in the stable AP group, respectively, to day 5 and all later time points (*p* < 0.04 for all) (Figure 1(a)).

#### 3.1.2. Nucleosomes (DNA-Histone Complexes)

Levels of nucleosomes were significantly higher in the STEMI group compared to the stable AP group at 3 and 12 hours (*p* < 0.03 for both) (Figure 1(b)).

No significant overall change or change from 3 hours to later time points was observed in the STEMI group. In the stable AP group, levels declined from baseline until day 3 (*p* < 0.05 for all), although no significant overall change was observed (Figure 1(b)).

### 3.2. The Time Profile of MPO

No difference between the groups was observed for levels of MPO at any time point (Figure 1(c)).

In both groups, a significant overall change was observed (*p* < 0.01) with declining levels from 3 hours to all later time points (*p* < 0.01 for all). Within the stable AP group, a significant increase from baseline to 3 hours was observed, sustained elevated at days 1, 3, 5, and 7 (*p* ≤ 0.03 for all) (Figure 1(c)).

### 3.3. Correlations between Markers of NETs and Neutrophil Proteins

In the total cohort, dsDNA and nucleosome levels intercorrelated significantly at the majority of time points (Table 2). Levels of MPO did not correlate with either dsDNA or nucleosome levels beyond a negative correlation to nucleosomes at 3 hours (*r* = −0.43, *p* = 0.02). Previously, we have investigated the time profile of pentraxin 3 (PTX3), another neutrophil granule protein and thus potential NETs component in the same cohort and observed that PTX3 levels were elevated shortly after PCI [23]. PTX3 levels did not correlate significantly with either dsDNA or nucleosomes at any time point (data not shown).

### 3.4. Correlations with Total Leukocyte Count

In the total cohort, levels of dsDNA correlated positively with total leukocyte count at the majority of time points while the nucleosomes correlated to a lesser degree. Levels of MPO correlated with total leukocyte count only at day 14 (Table 3). The correlations were less obvious when analyzing the groups separately (data not shown), although the time profile of total leukocyte count in both groups moderately imitated the pattern of dsDNA (Figure 1(d)).

### 3.5. Correlations with Myocardial Injury

**Figure 2:**
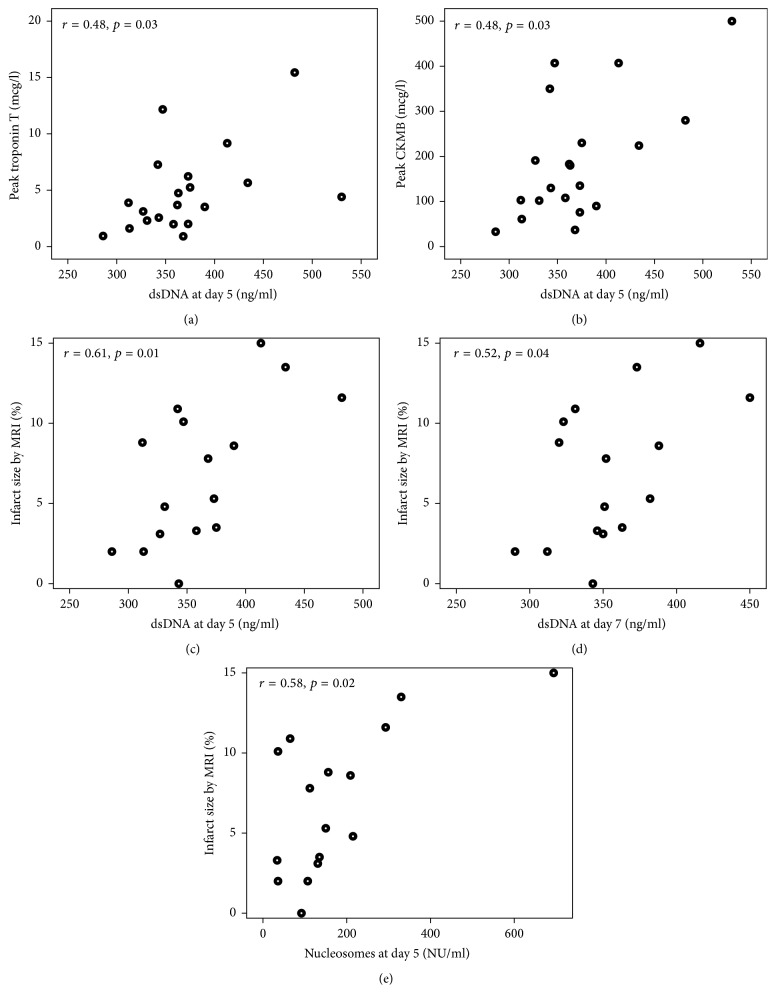
Correlations between NETs markers and indices of infarct size. dsDNA: double-stranded deoxyribonucleic acid. CKMB: creatine kinase MB. MRI: magnetic resonance imaging.

Levels of dsDNA correlated positively with peak levels of troponin T and CKMB at day 5 (*r* = 0.48, *p* = 0.03 for both), while day 5 and 7 levels correlated with infarct size assessed by MRI (*r* = 0.61, *p* = 0.01 and *r* = 0.52, *p* ≤ 0.04, resp.) (Figures 2(a)–2(d)). Also, nucleosome levels at day 5 correlated with infarct size by MRI (*r* = 0.58, *p* = 0.02) (Figure 2(e)), while levels of MPO were not correlated with any indices of myocardial injury. Either dsDNA, nucleosome, or MPO levels correlated with LVEF at any time point (data not shown).

## 4. Discussion

The main findings of this explorative, hypothesis generating study were as follows: (1) the two circulating surrogate markers of NETs, dsDNA and nucleosomes, were higher in patients with STEMI compared to patients with stable AP shortly after PCI, and levels were to a certain degree associated with indices of myocardial injury; (2) dsDNA and nucleosome levels decreased after PCI also in patients with stable AP, indicating that transient coronary ischemia might be associated with production of these NETs markers; (3) circulating levels of MPO did not reflect levels of dsDNA or nucleosomes and were not affected by the STEMI, but apparently by the PCI procedure itself.

Although circulating DNA has been reported to be higher in AMI than in both stable AP and healthy controls [11–15], no studies have to the best of our knowledge reported on the detailed time profile of NETs surrogate markers during STEMI and stable AP. The observed elevated levels of dsDNA and nucleosomes in patients with STEMI shortly after PCI are noticeable and may reflect enhanced neutrophil cell activation, myocardial cell necrosis with subsequent release of nuclear content, or a combination of more events. As strong correlations between dsDNA and nucleosome levels obtained in the subacute phase following PCI and infarct size measured by MRI after 6 weeks were observed, these potential markers of NETs could reflect myocardial cell necrosis directly. The possibility that they also represent neutrophil cell activation is nevertheless present, as strong correlations between circulating total leukocyte count and both dsDNA and nucleosome levels were observed at most time points and because the time profile of total leukocyte count imitated the time profile of dsDNA. Neutrophils have further previously been suggested to be the major source of circulating levels of both dsDNA and nucleosomes [10, 24]. As NETs probably hold prothrombotic properties like platelet activation [18], coagulation activation by factor XII and tissue factor, tissue factor pathway inhibitor (TFPI) suppression [19, 20] and inhibition of fibrinolysis [21], a role in coronary thrombosis would be plausible. As the present sample size was limited and the infarcts were relatively small, possible linkages between the assessed markers of NETs and coronary thrombosis need further exploration.

Elevated levels of dsDNA and nucleosomes shortly after PCI were not limited to the STEMI group, as decreasing levels of both markers also were observed in the stable AP group throughout the study period. In line with our observations, levels of both dsDNA and nucleosomes have previously been reported to associate with the severity of coronary atherosclerosis [10, 12], indicative of a link between AP with coronary ischemia and stimulated production of NETs (so-called NETosis). The pathophysiological properties of NETs in atherosclerosis and thus potentially also in AP have not been explored in detail, but have in murine models been suggested to include stimulation and migration of various innate and adaptive immune cells into the atherosclerotic plaque, partly through macrophage-mediated cytokine release [16, 25] and endothelial dysfunction mediated through metalloproteinases [26]. Given the prothrombotic properties of NETs, it would also be intriguing to speculate whether NETs participate in microthrombosis following intraplaque hemorrhage, a well-established cause of atherosclerotic plaque progression [27].

Levels of MPO, a well-known neutrophil granule protein and a proposed component of NETs [7, 28], were not reflected in neither dsDNA nor nucleosome levels. If dsDNA and nucleosomes indeed are part of NETs, this observation suggests that circulating MPO were mainly not NETs-derived. The lack of association between MPO and dsDNA/nucleosomes is consistent with that of another neutrophil granule protein, PTX3 [23], suggesting that despite release from neutrophil granules upon neutrophil cell activation, these two proteins did not enter circulating blood simultaneously with dsDNA and nucleosomes and did thus presumably not reflect NETosis in this cohort. Further, while MPO levels were not influenced by the STEMI, increased levels shortly after PCI in the stable AP group may be indicative of a direct effect of the PCI procedure per se. The mechanistic explanation for this apparent PCI-effect is unclear. Lastly, MPO was not correlated with any indices of myocardial injury or left ventricular function in contrast to the previously reported adverse properties of MPO in left ventricular remodeling after AMI [29, 30]. Again, limited sample size and small infarct sizes may have influenced these results.

The discovery of markers of NETs in atherothrombosis is of particular interest as NETs could be subject to therapeutic manipulations, that is, through administration of enzymes able to dissolve the NET structure like deoxyribonucleases (DNAses) or through inhibition of peptidylarginine deiminase 4 (PAD4), an essential enzyme in NETosis. Although in vivo experiments in humans have not been performed so far, adding DNAse to standard thrombolysis (with t-PA) has been shown to accelerate thrombus lysis in human ex vivo thrombi [8] and to reduce myocardial no-flow area, infarct size, and ischemia-reperfusion-induced left ventricular remodeling in rats [31]. Likewise, PAD4 inhibition has been shown to reduce NETosis in both murine and human neutrophils and further to directly interfere with atherosclerotic burden in mice [17, 32, 33].

## 5. Limitations

Beyond limited sample size and small infarctions as previously described, the hypothesis generating nature of this study carries methodological limitations, as the high CV for the nucleosome analysis. To what extent circulating levels of dsDNA and nucleosomes are specific for NETs is further not clear. Moreover, aspirin, heparin, and statins have all been reported to suppress or destabilise NETs in murine and human models [18, 34–37], which could explain falling levels of dsDNA and nucleosomes in both groups, as well as lower levels generally in the AP group. Finally, as differential counts for neutrophils, the major source of dsDNA [24], were not available, our analyses are based on total leukocyte counts. These are though to a high degree representative for circulating neutrophils.

## 6. Conclusions

High levels of the NETs markers dsDNA and nucleosomes were observed in patients with STEMI shortly after revascularisation and did partly reflect infarct size. The decline over time, also observed in AP patients, may indicate that transient coronary ischemia also stimulates release of NETs markers. Together, these observations may imply roles for NETs in atherothrombosis.

## Figures and Tables

**Table 1:** Clinical characteristics of the study population.

	Acute MI group *n* = 20	Stable AP group *n* = 10	*p*
Age (yrs)	60 (54–68)	64 (54–71)	ns
Female gender	5	1	ns

Established CAD	0	7	<0.01
Hypertension	7	4	ns
Diabetes mellitus (type 1 or 2)	2	2	ns
Previous or current smoking	10	3	ns

Medication at study inclusion			
Acetylsalicylic acid	1	7	<0.01
Clopidogrel	0	0	ns
ACE/AT II antagonists	5	2	ns
Beta-blocker	2	4	0.02
Aldosterone antagonist	0	0	ns
Insulin	0	0	ns
Diuretics	0	0	ns
Statins	1	7	<0.01

Medication at hospital discharge			
Acetylsalicylic acid	20	10	ns
Clopidogrel	20	9	ns
ACE/AT II antagonists	11	2	ns
Beta-blocker	18	6	ns
Aldosterone antagonist	0	0	ns
Insulin	0	0	ns
Diuretics	1	0	ns
Statins	20	10	ns

Indices of infarct size and left ventricular function			
Peak troponin T (*μ*g/L)	3.8 (2.1–6.1)		
Peak CKMB (*μ*g/L)	158 (93–268)		
Infarct size (MRI, %)^a^	6.6 (3.2–10.7)		
LVEF (MRI, %)^a^	58 (53–66)		

Values are given as numbers or medians (25–75 percentiles) unless otherwise stated. CAD: coronary artery disease, defined as previous angina, *Q*- or non-*Q* infarction, percutaneous intervention (PCI), or coronary artery bypass grafting (CABG). ACE/AT II antagonists: angiotensin converting enzyme/angiotensin II antagonists. CKMB: creatine kinase MB. LVEF: left ventricular ejection fraction. ^a^Measured 6 weeks after study inclusion.

**Table 2:** Correlation between levels of dsDNA and nucleosomes at corresponding time points in the total study population (*n* = 30).

	*R*	*p*
BL	0.17	ns
3 hours	0.51	<0.01
12 hours	0.63	<0.01
Day 1	0.40	0.03
Day 3	0.28	ns
Day 5	0.53	<0.01
Day 7	0.21	ns
Day 14	0.50	<0.01

*R*: Spearman rho. BL: baseline.

**Table 3:** Correlation between markers of NETs, MPO, and total leukocyte count at corresponding time points in the total study population (*n* = 30).

	dsDNA		Nucleosomes		MPO	
	*R*	*p*	*R*	*p*	*R*	*p*
Baseline (*n* = 10)	0.61	ns	0.13	ns	0.06	ns
3 hours	0.42	0.03	0.37	0.05	−0.04	ns
12 hours	0.37	0.05	0.30	ns	0.36	ns
Day 1	0.22	ns	0.45	0.01	0.07	ns
Day 3	0.57	<0.01	0.51	<0.01	0.17	ns
Day 5	0.54	<0.01	0.59	<0.01	0.36	ns
Day 7	0.49	<0.01	0.18	ns	0.09	ns
Day 14	0.39	0.04	0.21	ns	0.47	0.01

*R*: Spearman rho. BL: baseline.
